# Quantification of Global DNA Methylation in Canine Mammary Gland Tumors *via* Immunostaining of 5-Methylcytosine: Histopathological and Clinical Correlations

**DOI:** 10.3389/fvets.2021.628241

**Published:** 2021-02-25

**Authors:** Luiz Roberto Biondi, Marcello Vannucci Tedardi, Luciana Boffoni Gentile, Patricia Pereira Costa Chamas, Maria Lucia Zaidan Dagli

**Affiliations:** ^1^Department of Small Animal Internal Medicine, School of Veterinary Medicine, Santos Metropolitan University, Universidade Metropolitana de Santos (UNIMES), São Paulo, Brazil; ^2^Department of Pathology, School of Veterinary Medicine and Animal Science, University of São Paulo, São Paulo, Brazil

**Keywords:** mammary adenocarcinoma, methylation, 5mC, epigenetic, epigenome

## Abstract

Mammary tumors are the most prevalent neoplasms in non-neutered female dogs, with genetic and epigenetic alterations contributing to canine mammary carcinogenesis. This study quantified global DNA methylation in 5-methylcytosine (5mC)-immunostained canine mammary tumor samples and established histopathological and clinical correlations. A total of 91 formalin-fixed paraffin-embedded mammary tumor samples from female dogs were retrospectively selected and subjected to immunohistochemistry using an anti-5mC mouse monoclonal antibody. We evaluated 5mC+ stained nuclei of neoplastic epithelial cells in canine mammary glands to obtain semiquantitative histoscores based on staining intensity. Survival rates were estimated based on owners' or veterinary records. Histological samples comprised 28 and 63 benign and malignant canine mammary gland tumors, respectively. Results revealed significant differences between global DNA methylation patterns when mammary samples were categorized as benign or malignant (*p* = 0.024), with hypomethylated patterns more prevalent in malignant tumors and those with higher relapse behavior (*p* = 0.011). Of note, large diameter (>5 cm) tumors revealed a lower methylation pattern (*p* = 0.028). Additionally, we found non-statistically significant differences when tumors were grouped by histopathological characteristics, clinical parameters, or survival. These findings propose global DNA methylation assessment as a promising tool for detecting canine mammary tumors with relapse propensity.

## Introduction

Mammary tumors are the most prevalent neoplasm in non-neutered female dogs (1) representing ~50% of tumor diagnoses (2). The risk of developing mammary tumors is associated with hormone levels, breed susceptibility, age, diet, and obesity (3). Although numerous studies investigated genetic alterations in canine mammary cancers (4), including underlying molecular signatures (5), few studies have investigated epigenetic alterations in these tumors (6, 7). Moreover, to the best of our knowledge, none of the former studies have evaluated the global DNA methylation status.

Cancer development is strongly associated with specific genetic alterations. While epigenetic changes (including DNA methylation, histone modification, and microRNA expression) are independent of hereditary patterns or gene mutations, these changes are also critical to neoplastic initiation and progression. Indeed, several different epigenetic changes have been identified that contribute to the development and maintenance of the neoplastic phenotype (8, 9). In the mammalian genome, methylation occurs at cytosine bases located at the 5′ end of guanine bases, promoting the formation of CpG dinucleotide islands. These CpG dinucleotide islands play a central role in transcriptional repression (when most CpG dinucleotides in an island are methylated). Thus, methylation and demethylation (catalyzed by methyltransferases and demethylases, respectively) are central to transcriptional regulation (9–11). Oncogene activation and inactivation of tumor suppressor genes represent critical steps at every tumorigenesis stage. Furthermore, DNA methylation is central to transcriptional repression once the majority of CpG dinucleotides in the mammalian genome are methylated, except for CG-dense regions located around transcriptional start sites, known as CpG islands. Thus, methylation, which occurs at cytosine bases (C), located 5′ to guanine (G) bases to form CpG dinucleotide islands, can be affected by loss or gain of DNA methyltransferases or demethylases. From an epigenetic standpoint, global DNA hypomethylation can result in overexpression of growth factors, alterations in DNA repair enzymes, and/or loss of genomic stability. Its counterpart, aberrant hypermethylation, can silence tumor suppressor gene promoter regions. These are both well-established mechanisms through which cancer cells may acquire critical features on their pathway to transformation (9–12). In addition, like genetic mutations, epigenetic alterations can be mitotically inherited, resulting in a rapidly growing cancer cell population by conferring advantages to tumor cells, resulting in uncontrolled growth (13, 14).

Global DNA methylation can be studied using several methods, including methylation-sensitive restriction endonucleases followed by analysis of the obtained fragments, and hydrolysis of genomic DNA followed by specific detection and quantification of 5-methylcytosine (5mC) content (15). However, these techniques require DNA extraction and are not compatible with the simple observation of cells and neoplastic tissues. Therefore, an immunochemical approach using monoclonal antibodies that recognize a methyl group on carbon number 5 of cytidine (anti-5mC antibodies) may be more suitable for investigating *in situ* DNA methylation. Moreover, this method allows computer-assisted quantification of global methylation to be performed on interphase nuclei in several cell types and on a cell-by-cell basis using microscopy (16). Piyathilake et al. (17) used a radiolabeled methyl incorporation assay and immunohistochemistry to assess global DNA methylation in squamous cell carcinoma in the human lung and highlighted the advantages of the latter technique, which was superior in demonstrating statistically significant differences between tumor and healthy tissues. More recently, using anti-5mC antibodies to analyze the bone marrow biopsies of patients with myelodysplastic syndrome (in which DNA methylation plays a pathogenic role), researchers have shown this immunohistochemistry assay to be a cost-effective and powerful tool for the prediction of overall survival (18).

As mammary tumors are morphologically complex, with the involvement of numerous patterns and cell types, using an immunohistochemical method associated with microscopic quantification appears more appropriate for evaluating global DNA methylation in these samples. Furthermore, we have previously used this method to evaluate global DNA methylation status in mast cell tumors (19) and lymphomas (20). Therefore, in the present study, we evaluated global DNA methylation in canine mammary gland tumors using immunohistochemistry and correlated our findings with tumor diagnosis and clinical outcomes.

## Materials and Methods

### Case Origin and Data Collection

The study protocol was approved by the Committee on Ethics on the Use of Animals from the School of Veterinary Medicine and Animal Science of the University of São Paulo (No. 1823251013/2014) and by the Human Ethical Committee of Santos Metropolitan University (No. CAAE 10105019.2.0000.5509). Canine mammary tumors were retrieved from the archives of the Veterinary School of the Santos Metropolitan University, UNIMES (Santos, Brazil). The samples were originally obtained from canine females undergoing therapeutic mastectomy over 5 years. A total of 91 formalin-fixed paraffin-embedded (FFPE) mammary gland tumor samples from female dogs were retrospectively included in the study.

### Inclusion Criteria

Patients were eligible for this retrospective study when their records included a histologic diagnosis of benign neoplastic mammary lesions or malignant neoplastic mammary lesions. The inclusion criteria were as follows: (1) a minimum follow-up period of 18 months (follow-up intervals of 30, 90, 180, 360, and 540 days after mastectomy, including reexamination and thoracic radiographs); and (2) no previous history of mastectomy and/or history of neoadjuvant chemotherapy. The clinical-stage was obtained according to the World Health Organization TNM classification (21). Distant metastasis was investigated by three-way thoracic radiography and abdominal ultrasound. Lymph node involvement was determined by cytologic and histologic examinations.

### Tissue Processing for Histological Grade Determination and Immunohistochemistry

FFPE mammary tissue fragments were sliced into 5 μm-thick sections, mounted on glass slides, and stained with hematoxylin and eosin for classification according to the histological type. The diagnosis was conducted by three different pathologists (LRB, LBG, and an external private pathology laboratory) based on previously defined classification criteria (21), and histological grading was based on previously described criteria (22, 23).

Immunohistochemistry to detect 5mC involved dewaxing silanized slides containing 5 μm-thick tumor slices using xylene and progressive dehydration in alcohol. Antigen retrieval was performed with citrate buffer (pH 6.0) in a microwave oven at 97°C for 15 min, after which endogenous peroxidases were inactivated with 30% oxygen peroxide in methanol. The slides were then incubated with a primary antibody (ab10805 anti-methylcytosine (5-mC) antibody [33dD3] Abcam, Cambridge, UK) diluted 1:150 and kept at 4°C overnight. After rinsing, the slides were incubated with secondary antibody (LSAB Kit + System-HRP; K0679; DakoCytomation, Glostrup, Denmark) at room temperature (25°C) for 30 min. Samples were stained with a diaminobenzidine chromogen (Dako Corp., Carpinteria, CA, USA) and counterstained with hematoxylin. Between each step, slides were rinsed three times with 1 × phosphate-buffered saline (PBS) or 1 × PBS with Tween 20 (Dako Corp.), according to the manufacturer's instructions. Validated positive tissues (canine mast cell tumor and lymphma) obtained from previous studies performed in our laboratory were used as controls in order to check the quality of the antibody batch in use. Negative controls were obtained by replacing the primary antibody with a control match-isotype antibody (IgG1).

### Data Acquisition and Histoscore (H-Score) Quantification

Immunohistochemical sections were evaluated under an optical microscope (NIKON, Tokyo, Japan) over 10 high-power fields, with the images recorded using the Image-Pro Plus system (v.4.5.0.29; Media Cybernetics, Rockville, MD, USA). The goal was to achieve a minimum of 500 cells/tumor, as per previously described methods (16), and tumors with <300 cells were excluded (in summary, 15 samples had more than 300 cells and <400; 10 samples had more than 400 cells and <500 and 66 samples had between 500 and 1,475 cells). Mammary gland epithelial cells were considered 5mC+ if they presented nuclei with dark brown/gold coloration after nuclear staining. Slides were evaluated by two different examiners (LRB and PPCC), and the final score was determined based on the average of the counts. As there is no standardization for 5mC immunoreactivity for canine mammary tumors, quantification of immunohistochemistry results was based on the H-score, a semiquantitative method accepted by the American Society of Clinical Oncology (24). Nuclear staining intensity was first classified as 0 (no staining), 1+ (light staining), 2+ (intermediate staining), or 3+ (strong staining), for each cell in a fixed field, and the H-score was then obtained as [1 × (% cells 1+) + 2 × (% cells 2+) + 3 × (% cells 3+)] in a range from 0 to 300 (25). This study considered only epithelial cells for methylation patterns, in line with most recent global DNA methylation studies.

### Survival Analyses

**Figure 1:**
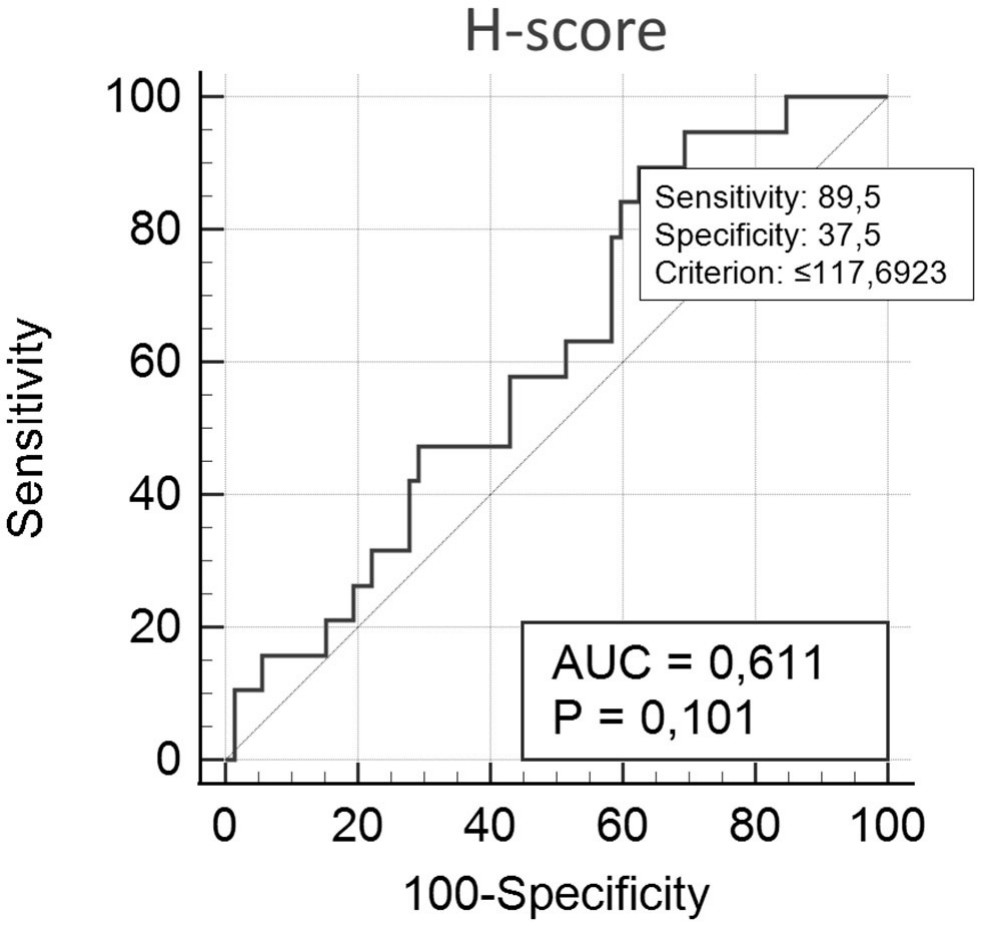
H-score, according to ROC analysis. Variables under analysis were the methylation score and the classification variable (death by mammary gland tumor). Cut-off value: <117.7. Sensitivity and specificity were determined as 89.5 and 37.5, respectively, with an area under the ROC curve of 0.611 (*p* = 0.101; *n* = 63).

For survival and other statistical analyses, scores were converted into two categorical variables (methylated or hypomethylated) either above or below (respectively) a cut-off value of 117.7 (e.g., borderline tumor value: 124.6094 = methylated), as obtained through receiver operating characteristic (ROC) curve analyzes, as per previously described methods (18). The variables under analysis were the methylation score and classification variable (death by mammary gland tumor), as shown in Figure 1.

Although prognosis was beyond the scope of this work, information concerning the survival rate was obtained *via* telephone conversation with animal owners or from medical records. The overall survival time was calculated from the date of mastectomy to the date of patient death. The Kaplan–Meier method was used to calculate overall survival time, and the log-rank test was used to identify factors associated with post-mastectomy survival. Live animals, mammary gland tumor-unrelated deaths, or missing animals due to lack of follow-up were removed for statistical purposes.

Other statistical analyzes included a normality test (Kolmogorov-Smirnov), unpaired *t*-test, and one-way analysis of variance (ANOVA). All analyzes were conducted using MedCalc® software (v.19.6.1), and a *p* < 0.05 was considered statistically significant.

## Results

Canine mammary gland tissue was obtained from 91 females of different breeds (average age at the time of surgery: 9.2 ± 2.6 years). Of these animals, 16 had been previously neutered, 6 were histopathologically diagnosed with a neoplastic invasion of inguinal or axillary lymph nodes, and 1 received a lung metastasis diagnosis upon radiographic examination during the follow-up.

Histologic diagnosis comprised 28 benign tumors (accounting for 30.8% of all tumors) and 63 malignant tumors (accounting for 69.2% of the tumors in the studied population). Of the malignant tumors, 52.4% (*n* = 33/63) were classified as grade I, 30.2% (*n* = 18/63) as grade II, and 17.5% (*n* = 12/63) as grade III. Two animals had clinical and histopathologic diagnoses consistent with inflammatory carcinoma with marked vasculogenic skin invasion. Table 1 summarizes the distribution of tumors according to the histologic diagnosis and grade.

**Table 1:** Neoplastic lesions distribution by histological type and grade.

**Histological description**	**Quantity and frequency %**	**Grade**		
		**I**	**II**	**III**
Benign mixed tumor	11 (10.0)	–	–	–
Adenoma—complex	5 (4.5)	–	–	–
Adenoma—simple tubular	4 (3.6)	–	–	–
Intraductal papillary adenoma	4 (3.6)		–	–
Fibroadenoma	1 (0.9)	–	–	–
Intraductal cystic-papillary adenoma	1 (0.9)	–	–	–
Nipple ductal adenoma	1 (0.9)	–	–	–
Tubulopapillary adenoma	1 (0.9)	–	–	–
**Subtotal**	**28 (30.8%)**	–	–	–
Complex carcinoma	12 (13.2)	9 (14.3)	3 (4.8)	–
Carcinoma—mixed	9 (9.9)	6 (9.5)	2 (3.2)	1 (1.6)
Carcinoma—simple tubular	11 (12.1)	4 (6.3)	4 (5.9)	3 (4.4)
Intraductal papillary carcinoma	5 (5.5)	3 (4.8)	2 (1.8)	–
Carcinoma in a complex adenoma	4 (4.4)	3 (4.8)	1 (1.5)	–
Carcinoma—simple cribriform	3 (3.3)		3 (4.4)	–
Carcinoma in a benign mixed tumor	3 (3.3)	2 (3.2)	1 (1.6)	–
Carcinoma—anaplastic	1 (1.1)	–	1 (1.5)	1 (1.5)
Carcinoma—solid	2 (2.2)	–	–	2 (3.2)
Comedocarcinoma	2 (2.2)	–	–	2 (3.2)
Inflammatory carcinoma:	2 (2.2)	–	–	–
*Carcinosarcoma*		–	1 (1.6)	–
*Carcinoma—simple tubular*		1 (1.6)	–	–
Carcinosarcoma	1 (1.1)	–	–	1 (1.6)
Carcinoma cystic-papillary	1 (1.1)	–	1 (1.6)	–
Carcinoma—micropapillary invasive	1 (1.1)	–		1 (1.6)
Carcinoma—simple tubulopapillary	1 (1.1)	1 (1.6)	–	–
Carcinoma and malignant myoepithelioma	1 (1.1)	1 (1.6)	–	–
Carcinoma in a simple tubular adenoma	1 (1.1)	1 (1.6)	–	–
Carcinoma in a simple tubulopapillary adenoma	1 (1.1)	1 (1.6)	–	–
Carcinoma in a fibroadenomatous dysplasia	1 (1.1)	–	–	–
Carcinoma—*in situ*	1 (1.1)	–	–	–
Ductal carcinoma *in situ*	1 (1.1)	1 (1.6)	–	–
Ductal carcinoma	1 (1.1)	–	–	1 (1.6)
**Subtotal**	**63 (69.2%)**	**33 (52.4)**	**18 (30.2)**	**12 (17.5)**
**Total**	**91 (100%)**		**63 (100%)**	

All animals underwent a full or partial mastectomy, and 23 showed disease relapses, including 13 with a new presentation of neoplastic disease in the remaining glands during follow-up, 10 of which had a recurrence in the surgical scar. Overall, 15 animals were alive, 27 animals had missed their follow-up appointments, and 49 animals succumbed. Among the deaths, 17 were attributed to mammary gland tumors, and the remaining were attributed to other causes, such as natural aging, heart or kidney failure, or euthanasia due to other tumor types or conditions.

**Figure 2:**
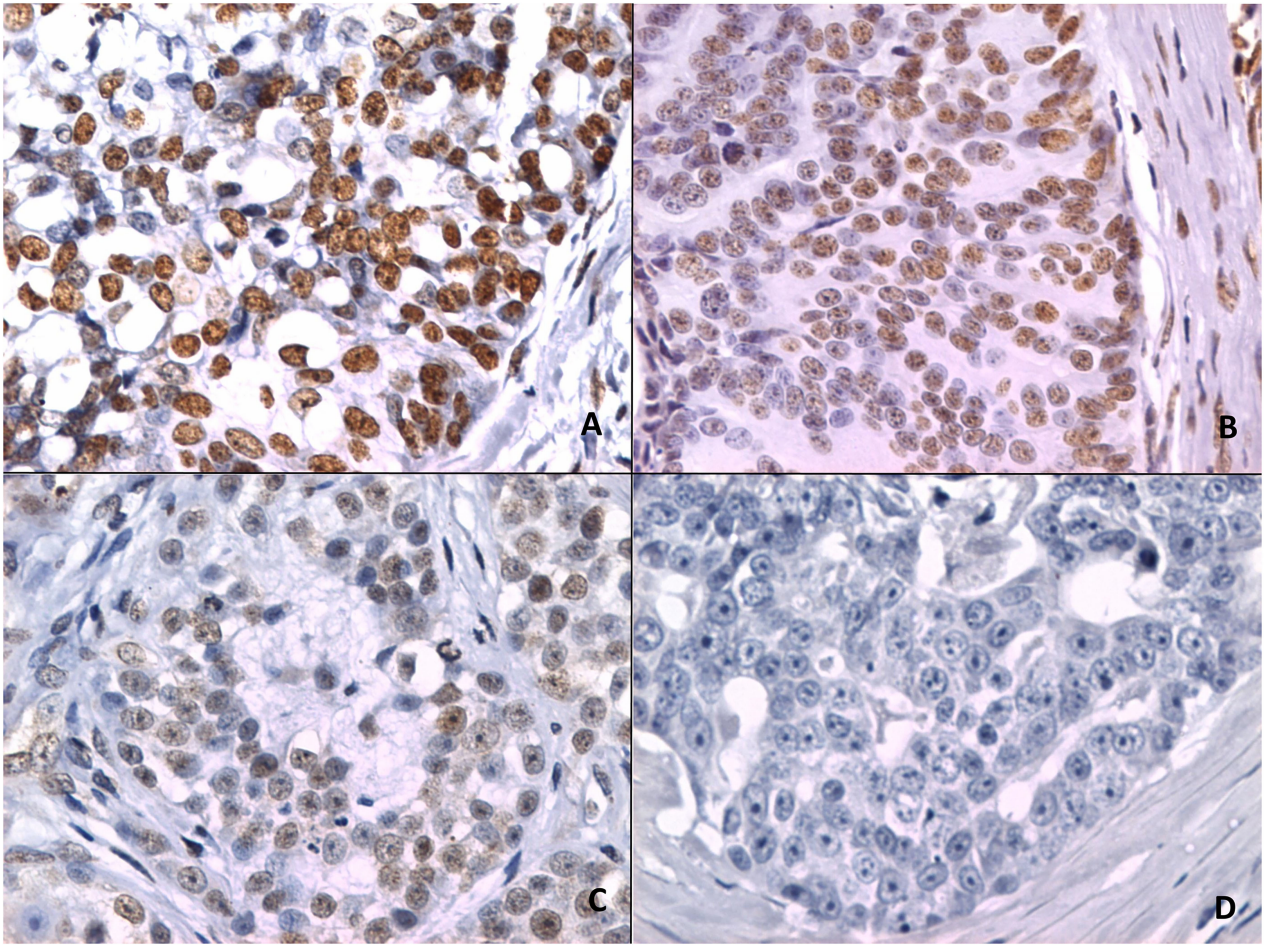
5mC immunohistochemistry. **(A)** Strong staining is characterized by intense dark brown color in a sample representing nipple ductal adenoma. **(B)** Moderate staining in a sample representing carcinoma in a simple tubular adenoma. **(C)** Weak staining with characteristic vacuolated nuclei in a sample with solid carcinoma. **(D)** A lack of staining in a sample representing comedocarcinoma.

**Figure 3:**
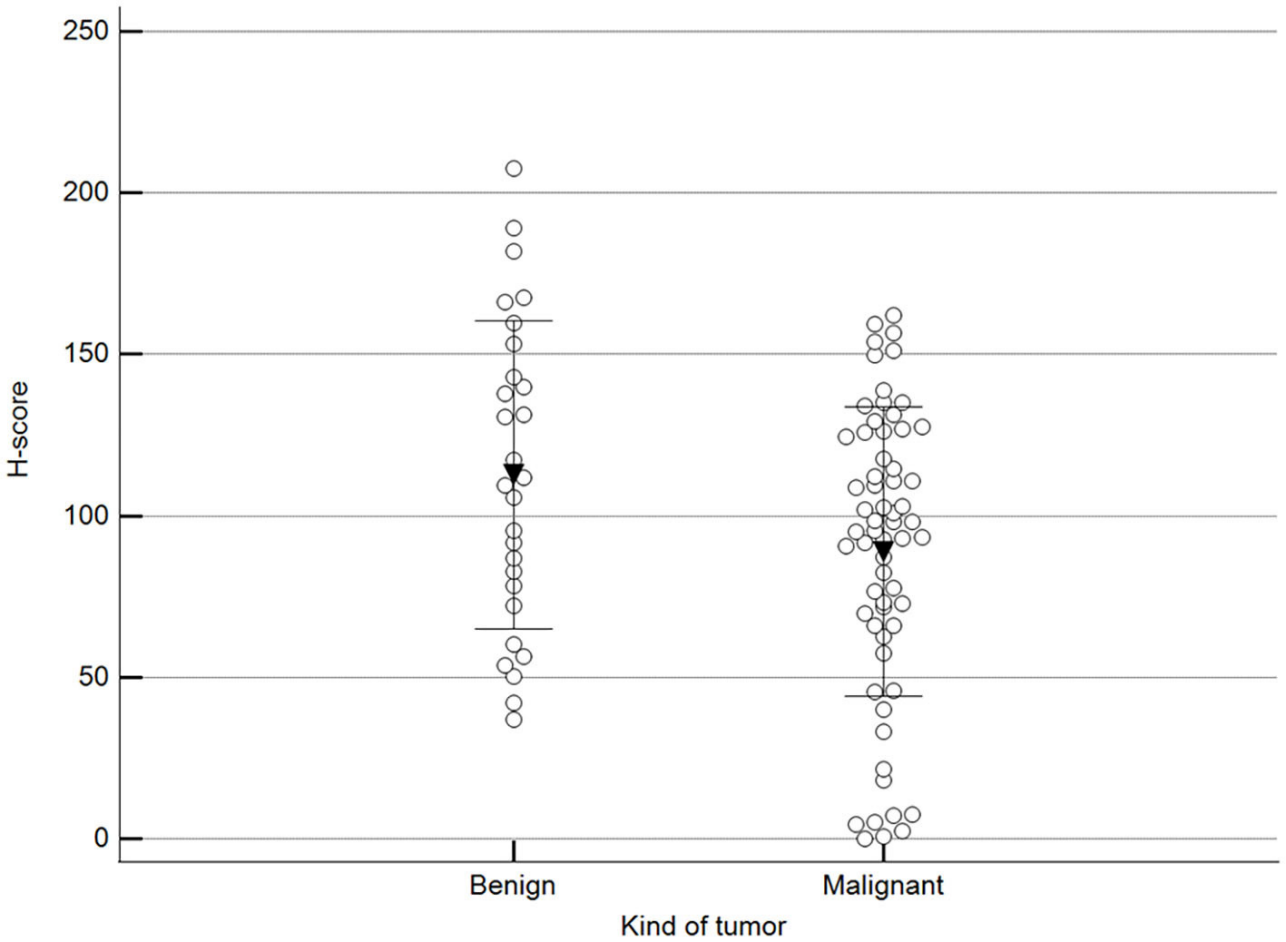
Scoring of methylation patterns according to 5mC immunohistochemistry. Tumors were grouped as benign (mean = 112.8; *n* = 28) or malignant (mean: 88.9; *n* = 63) (*p* = 0.024; Unpaired *t*-test). Dots represent each case; lines represent 1 standard deviation and the inverted triangle represents the value.

Figure 2 shows 5mC immunohistochemical staining of neoplastic mammary glands. Evaluation of 5mC immunoreactivity as a continuous variable revealed statistically significant differences that allowed the grouping of tumors as benign or malignant, with the latter being less methylated (Figure 3).

**Figure 4:**
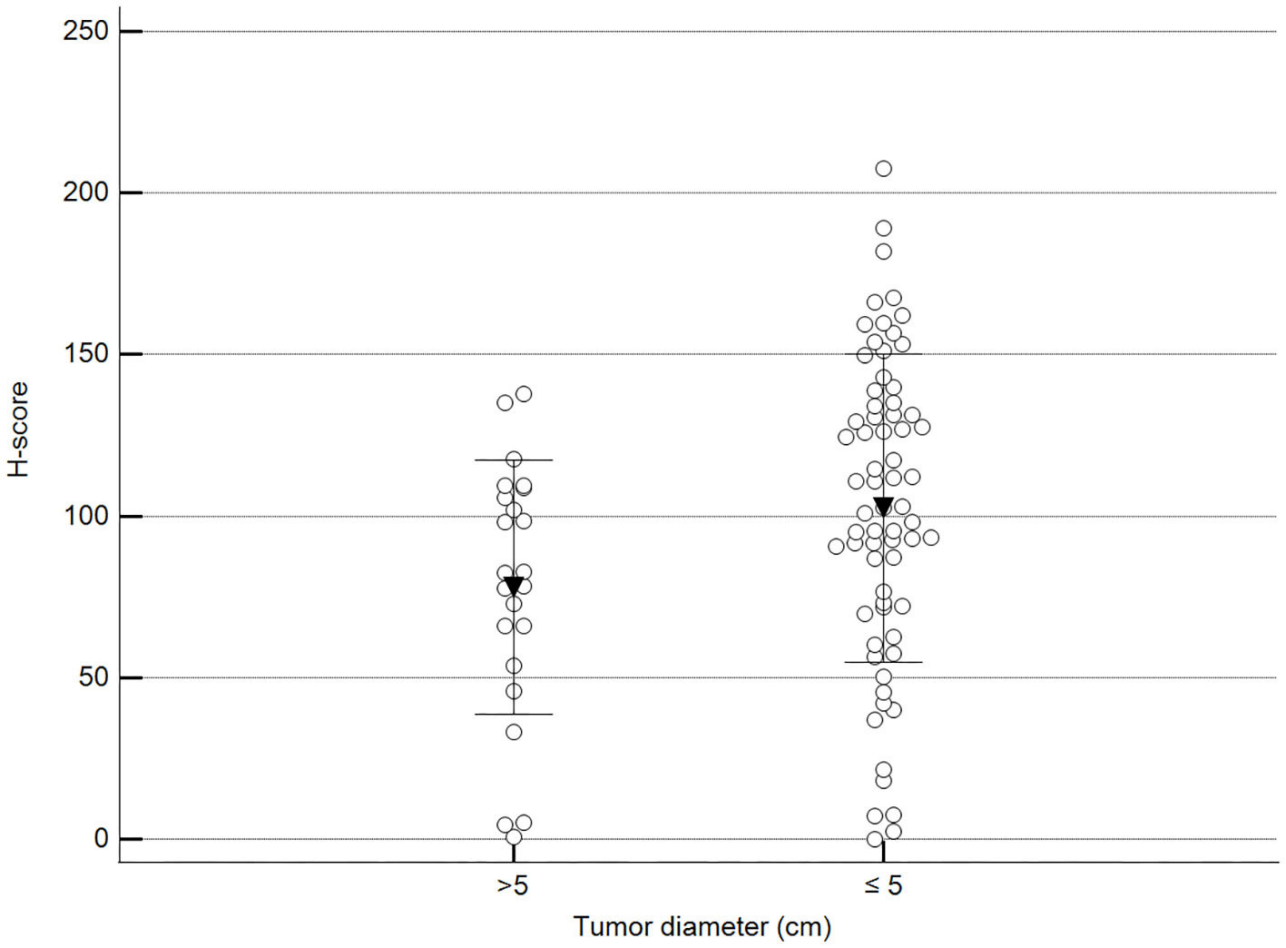
Methylation patterns based on 5mC H-score grouped by the largest tumor diameter. Tumors were grouped according to the largest tumor diameter: >5 cm (mean = 77.9; *n* = 23) and ≤5 cm (mean = 102.5; *n* = 68). Unpaired *t*-test (*p* = 0.028; *n* = 91). Dots represent each case; lines represent 1 standard deviation and the inverted triangle represents the value.

**Figure 5:**
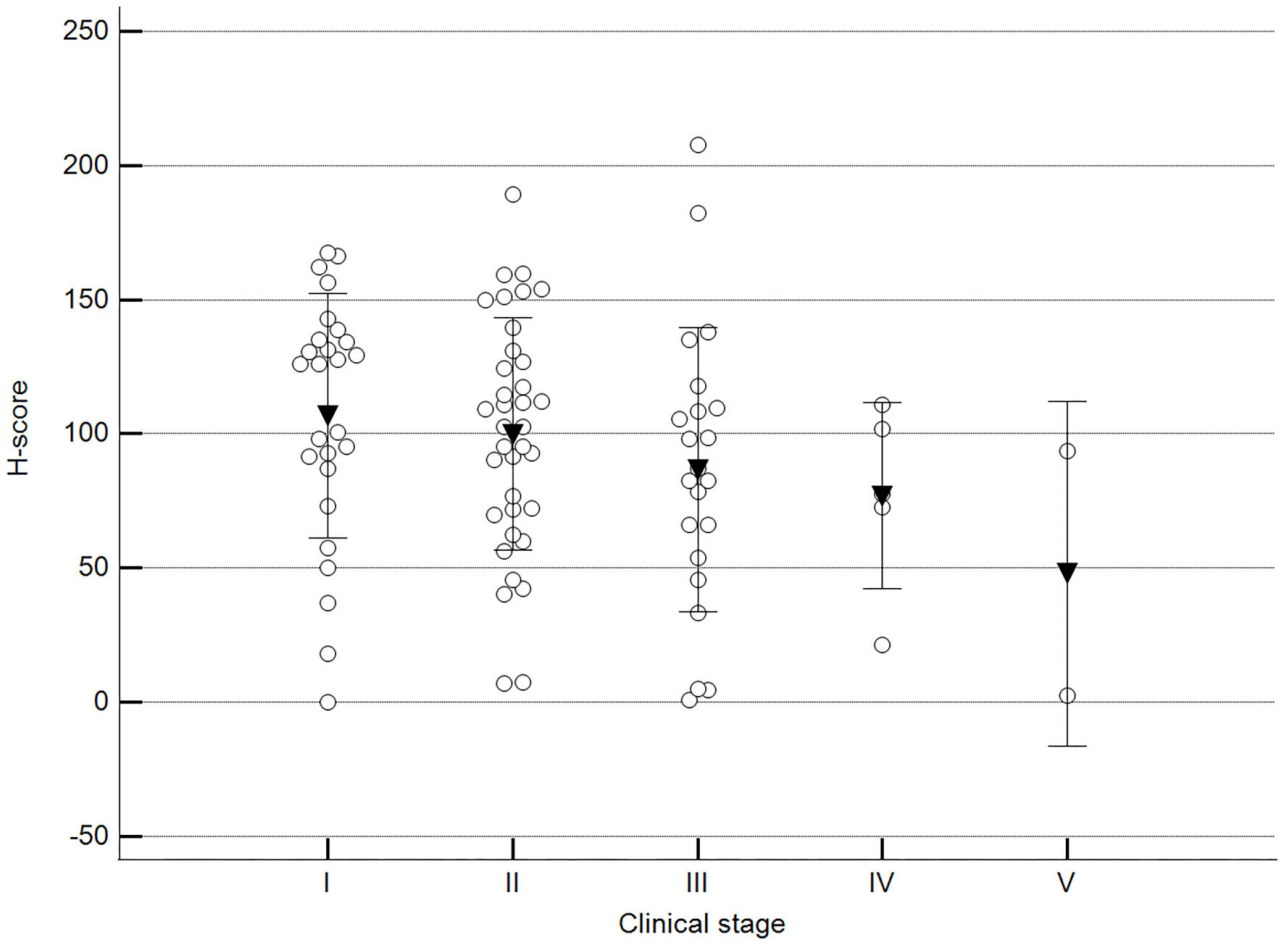
Methylation patterns based on 5mC H-score grouped by stage. Tumors were grouped as clinical stage I to V based on TNM grouping. One-way analysis of variance (*p* = 0.247; *n* = 91). Dots represent each case; lines represent 1 standard deviation, and the inverted triangle represents the value. Post-test not applicable.

**Figure 6:**
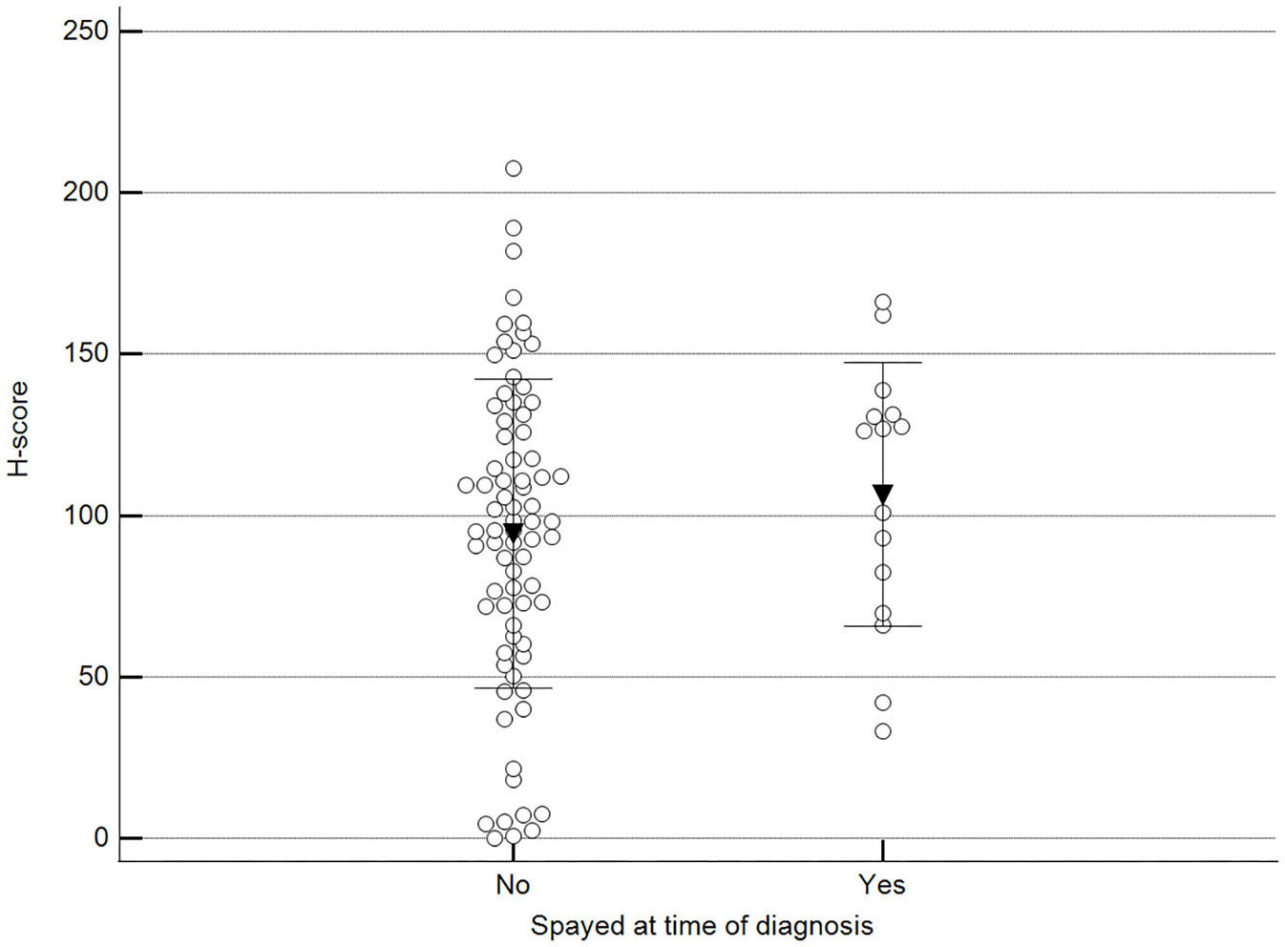
Methylation patterns based on 5mC H-score grouped by females already spayed at the time of diagnosis. Tumors were grouped as previously spayed = yes or no. Unpaired *t*-test (*p* = 0.359; *n* = 91). Dots represent each case; lines represent 1 standard deviation, and the inverted triangle represents the value.

**Figure 7:**
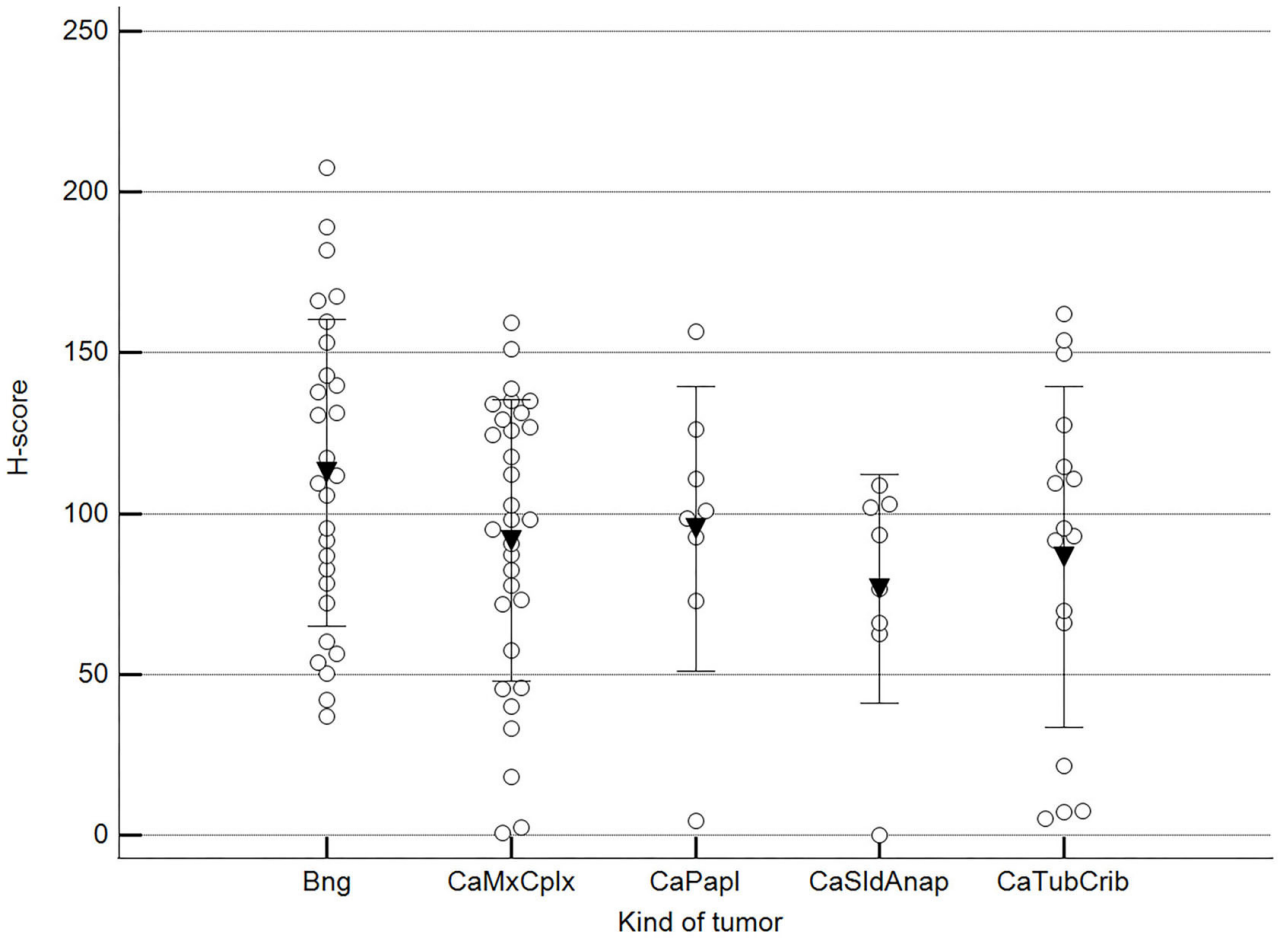
Methylation patterns based on 5mC H-score grouped by tumor histological group. Tumors were grouped as BNG, benign; CaMcCplx, carcinoma mixed and complex; CaPapl, carcinoma papillary; CaSldAnap, carcinoma solid and anaplastic; CaTubCrib, carcinoma simple tubular and cribriform. One-way analysis of variance (*p* = 0.206; *n* = 91). Dots represent each case; lines represent 1 standard deviation, and the inverted triangle represents the value. Post-test not applicable.

**Figure 8:**
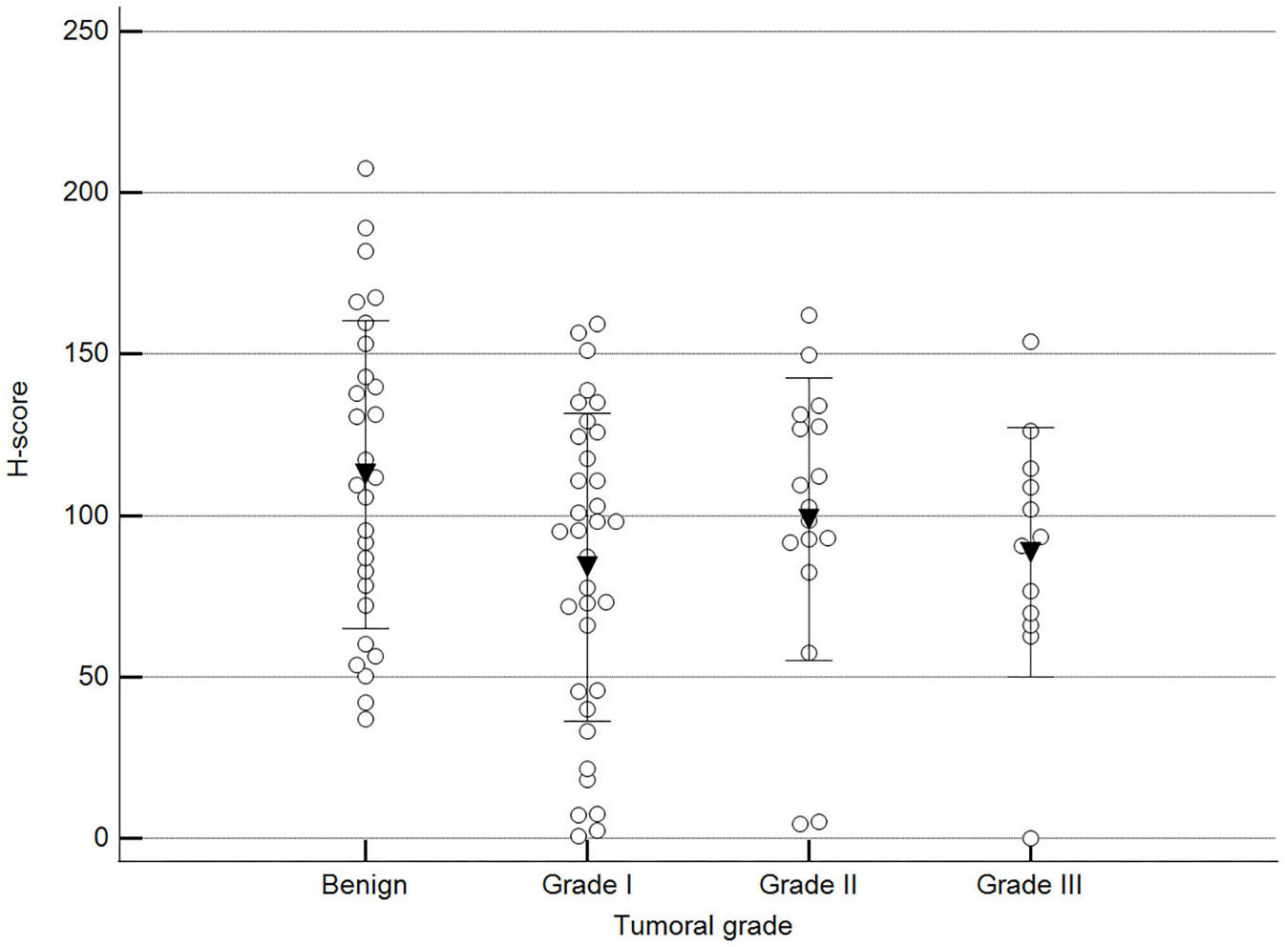
Methylation patterns based on 5mC H-score grouped by tumor histological grade. Tumors were grouped as benign or malignant grade I, II, or III. One-way analysis of variance (*p* = 0.100; *n* = 91). Dots represent each case; lines represent 1 standard deviation, and the inverted triangle represents the value. Post-test not applicable.

**Figure 9:**
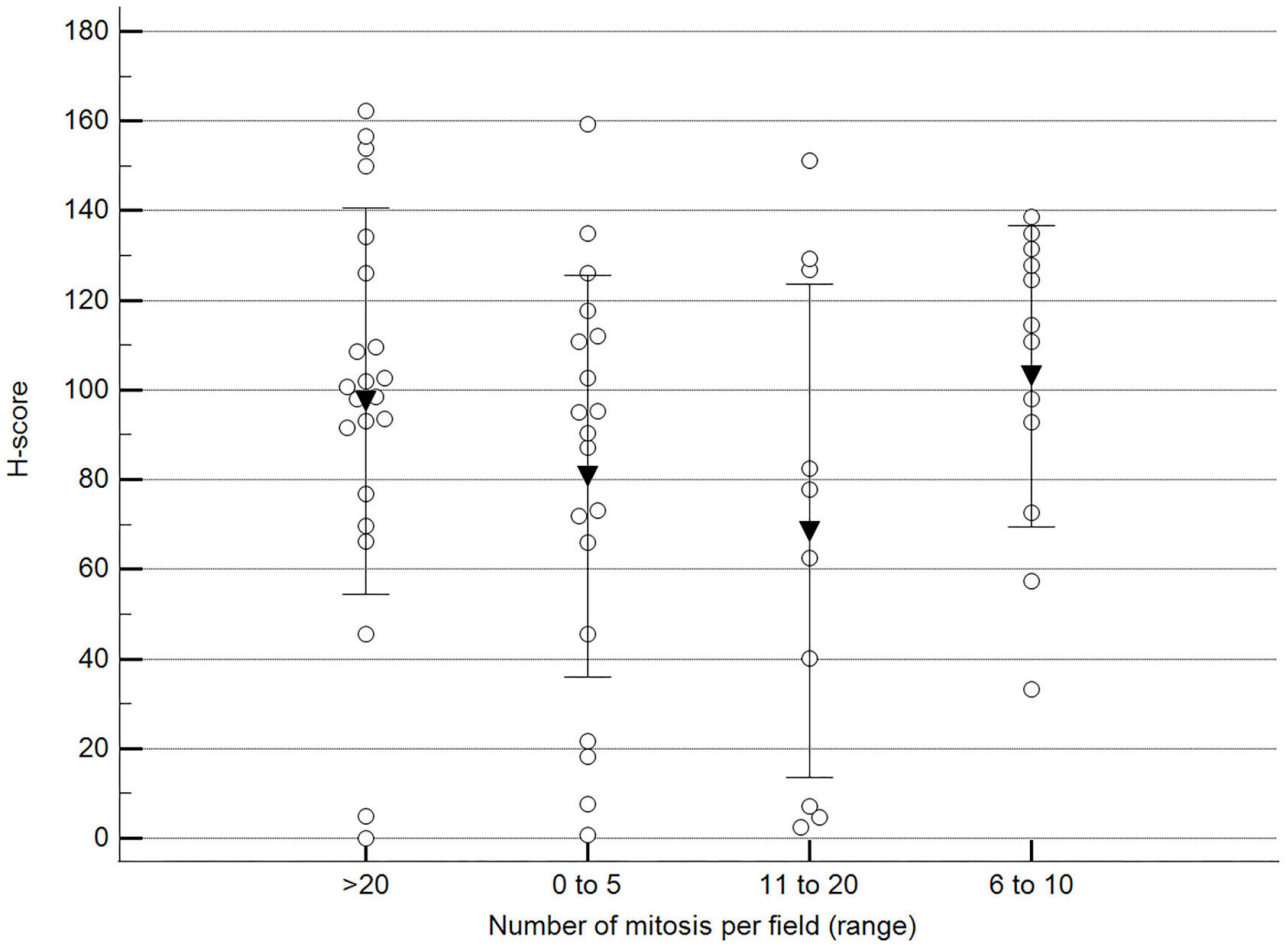
Methylation patterns based on 5mC H-score grouped by the number of mitoses per high-power field. Tumors were grouped according to the number of mitoses per field of 400× magnification. One-way analysis of variance (*p* = 0.196; *n* = 63). Dots represent each case; lines represent 1 standard deviation, and the inverted triangle represents the value. Post-test not applicable. Magnification: 400×.

We found statistically significant differences upon grouping methylation patterns by tumor size (Figure 4). However, we did not find statistically significant differences in methylation patterns when tumors were grouped by clinical parameters such as the stage (Figure 5), previous sterilization (Figure 6), or other tumoral characteristics, including histological type (Figure 7), tumor grade (Figure 8), and the number of mitoses in high-magnification fields (Figure 9).

**Figure 10:**
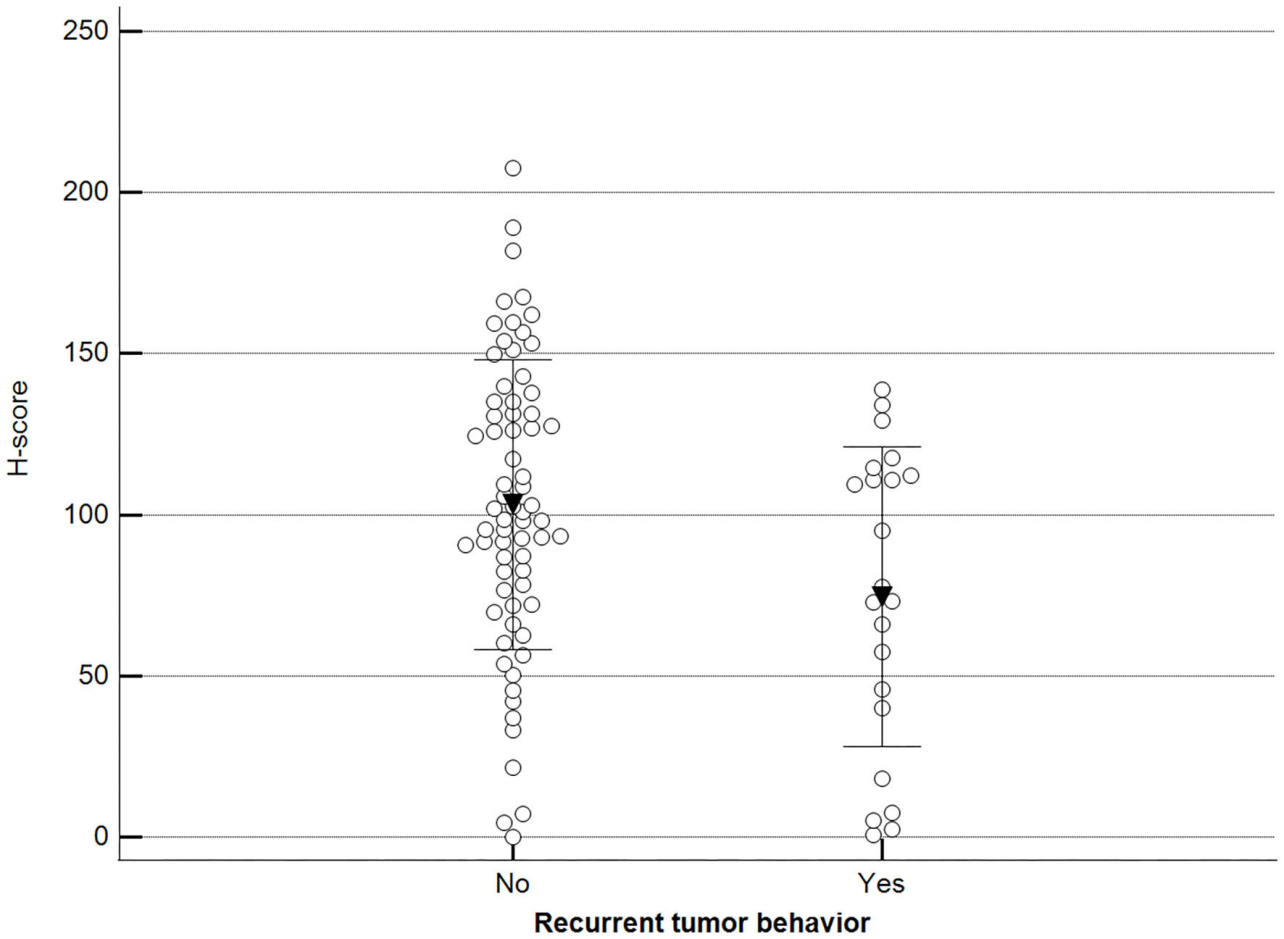
Methylation patterns based on 5mC H-score grouped by tumor relapse. Tumors were grouped as recurrence/new presentation = yes (mean = 74.5; *n* = 22) or no (mean = 103.2; *n* = 69). Unpaired *t*-test (*p* = 0.011; *n* = 91). Dots represent each case; lines represent 1 standard deviation and the inverted triangle represents the value.

Additionally, we observed a significant difference when malignant tumor scores were grouped by the occurrence of relapse (recurrence/new presentation) (Figure 10).

**Figure 11:**
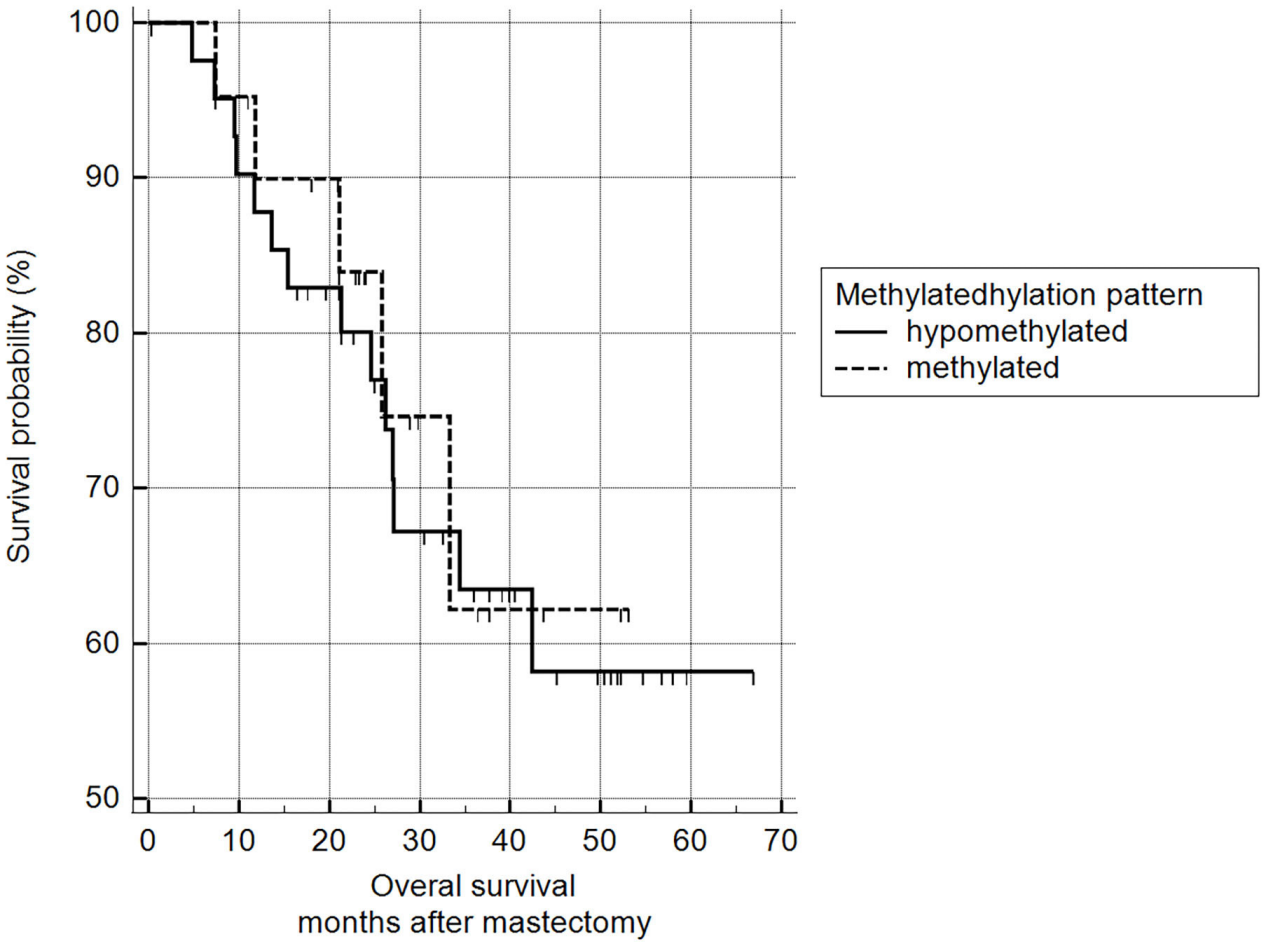
Survival analysis, according to 5mC H-score. Grouping according to hypomethylated or methylated tumor status revealed an H-score cut-off value of 117.7 (ROC) (*n* = 63). Mean survival of hypomethylated = 46.6 months and methylated = 47.0 months. Kaplan-Meier curve and log-rank test (*p* = 0.1594; *n* = 63).

5mC H-scores were subsequently transformed into a binary variable (methylated and hypomethylated) according to the ROC curve analysis and subjected to survival analysis. There was no statistically significant difference between the survival of animals with methylated malignant tumors and those with hypomethylated tumors (log-rank Mantel–Cox and chi-squared 1.9799 DF 1, *p* = 0.1594) (Figure 11).

## Discussion

In the present study, we retrospectively evaluated global DNA methylation status in canine mammary tumors and correlated these results with clinical parameters, survival, and tumor relapse. Few studies have reported the use of immunohistochemical techniques to evaluate global DNA methylation in canine neoplastic disease, and none have focused on global DNA methylation in mammary neoplasms. Moreover, to the best of our knowledge, this is the first study reporting global DNA methylation status in canine mammary tumors. The design of this study was based on the findings of Hernandez et al. (16), who evaluated global DNA methylation patterns in human colon cancer. They validated their immunohistochemistry results using an anti-5mC antibody and with immunoblotting results from genomic DNA samples of *Xanthomonas oryzae* DNA in which almost all the cytosine bases were replaced with 5mC. Furthermore, our results were in line with those reported by Morimoto et al. (19) and Epiphanio et al. (20). Their observations of global methylation patterns in canine mast cell tumors and canine lymphoma were obtained with the same antibody used herein.

We found that malignant mammary tumors demonstrated lower immunohistochemical staining scores compared with t benign tumors, similar to a previous study reporting immunohistochemical evaluation of overall DNA methylation patterns in mouse embryonic tissue and human prostate, breast, and colon tumor tissue (26). In that study, methylated CpG islands were converted to 5-hydroxymethylcytosine (5hC), and they reported high 5hC-staining levels in adult and embryonic tissues, closely related to the degree of cell differentiation and tissue organization. Whereas, low staining levels were observed in prostate carcinomas and breast and colon tumors.

Others studies demonstrated similar findings. One study assessed global DNA methylation profiles in paired normal and neoplastic colon tissue using an anti-5mC antibody (16). They described qualitative and quantitative differences between normal and neoplastic regions of paired colon tissues, with the latter being generally hypomethylated. Another study compared adjacent benign tissue and high and low degree prostate intraepithelial neoplasms (27). Furthermore, immunohistochemistry staining using an anti-5mC antibody and paraffin-embedded tissue in normal uterine cervix, benign lesions (including pre-neoplastic tissue), non-invasive malignant lesions, and samples of invasive squamous cell carcinoma of the uterine cervix, although the authors did not find a significant difference in global DNA methylation between normal, benign, and non-invasive malignant lesions, they reported a significant difference in methylation scores between the normal, benign, and non-invasive malignant lesion groups in comparison with the invasive squamous cell carcinoma group, which demonstrated a hypomethylated pattern (28). Due to the retrospective nature of the present study, there was a lack of adequate normal mammary tissue on the slides that could be used as a paired sample for the corresponding tumor lesion, a limitation of this work.

Interestingly, among the malignant tumors, the hypomethylated pattern was associated with tumors larger than 5 cm in diameter, suggesting that the loss of methylation may be associated with tumor progression, and particularly, with tumor proliferation. This finding is possibly supported by a previous report on the progression of mammary tumors in dogs (29) and breast tumor in women (30). In fact, a study that evaluated the methylation status in human hepatocellular carcinoma, with a sophisticated chip array technique in normal and tumoral liver tissue and tumors in progression, demonstrated that global hypomethylation in hepatocellular carcinoma was associated with chromosomal instability (31).

On the other hand, we also observed a correlation between 5mC immunoreactivity and tumor recurrence, with hypomethylated tumors demonstrating a propensity to relapse. In fact, a previous study reported this phenomenon in prostate tumor in men, and a similar observation was made in post-operative hepatocarcinoma (27, 32). Additionally, Mazzucchelli et al. (33) evaluated global DNA methylation profiles in papillary urothelial neoplasia of low malignant potential using immunohistochemistry and found a statistically significant difference between non-recurring and recurrent tumors, with the latter showing a hypomethylated pattern. As hypomethylation is implicated in genome instability and malignant transformation, we speculate that animals with hypomethylated mammary neoplasms could be at risk of tumor recurrence.

The methylation status and clinical correlations (such as staging, histological grade, and survival) observed here are comparable with previous studies showing no relationship between clinical prognostic factors and overall DNA methylation patterns between normal prostate tissue and prostate cancer samples (27, 34). In contrast with our results, some reports have demonstrated an ambiguous association between methylation status and poor overall survival. One study involving 5mC immunostaining of bone marrow biopsies from patients with myelodysplastic syndrome found significantly worse survival in patients with methylated bone marrow tissues (18). Another study assessed the methylation expression index (MEI) in tissue from women with invasive breast cancer. They found that decreased survival was associated with a low MEI index in 2,500 ER+ patients (35). Good et al. (36) used cell lines from triple-negative breast cancer patients to demonstrate worse overall survival in patients with hypomethylated tumors. These conflicting outcomes may be explained by the methodology adopted for investigating the cause of death. Additionally, as ours was a retrospective study, our data were limited to the quality of the information obtained from medical records or provided by owners, a limitation of our study.

Another study limitation was the inability to validate immunohistochemistry findings using other molecular techniques, as the material available was restricted to FFPE tissues. However, it should be noted that other techniques are biased, as they use complete portions of the triturated tumor tissues, including DNA from both tumor and stromal cells, thereby compromising the results. However, Piyathilake et al. (17) compared the *in vitro* radiolabeled methyl incorporation assay with immunohistochemical staining of the same tissue sections with a monoclonal antibody developed against 5mC. They concluded that immunostaining was a useful technique for evaluating global DNA methylation, especially when cancer-related methylation cannot be normalized to methylation of normal tissues or when the number of samples available for evaluation is small, as in the present study (17).

In summary, this is the first study describing global DNA methylation patterns in canine mammary tumors using 5mC immunostaining as an inexpensive and straightforward technique. Although no correlation between tumor type, grade, or clinical prognostic factors was observed, we effectively demonstrated that hypomethylation was more prevalent in malignant tumors and, particularly, among those with a diameter exceeding 5 cm. Our findings demonstrate the efficacy of this method for global DNA methylation assessment and suggest that DNA hypomethylation is associated with malignant tumors. This tool may help identify recurrent tumors and those with relapse potential, suggesting this marker is potentially useful in the prognostic evaluation of canine mammary neoplasms. However, proper validation of this technique should be performed using a gold standard quantitative technique.

## Data Availability Statement

The datasets presented in this study can be found in online repositories. The names of the repository/repositories and accession number(s) can be found at: https://1drv.ms/u/s!AsfWCRqEd3yHivplXdJRIUScP6GNBQ?e=Eo7Pa6.

## Ethics Statement

The animal study was reviewed and approved by Committee of Ethics on the Use of Animals (CEUA) of the School of Veterinary Medicine and Animal Science of the University of São Paulo. Written informed consent was obtained from the owners for the participation of their animals in this study.

## Author's Note

This study is part of the doctoral dissertation project by LB at the Graduate Program on Experimental and Comparative Pathology of the School of Veterinary Medicine and Animal Science of the University of São Paulo, Brazil.

## Author Contributions

LB and MD: conceptualization and manuscript writing. LB, LG, MT, and MD: methodology. LB: software. MD: grant acquisition and supervision. All authors contributed to the article and approved the submitted version.

## Conflict of Interest

The authors declare that the research was conducted in the absence of any commercial or financial relationships that could be construed as a potential conflict of interest.
